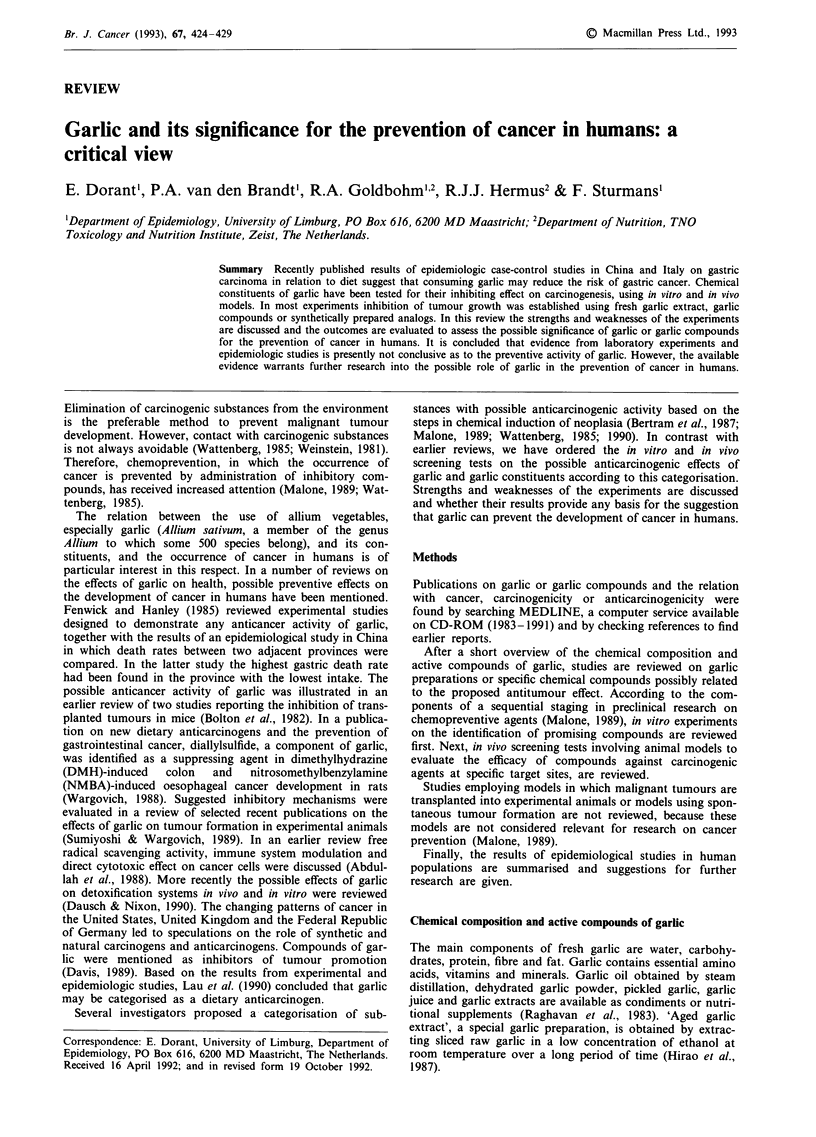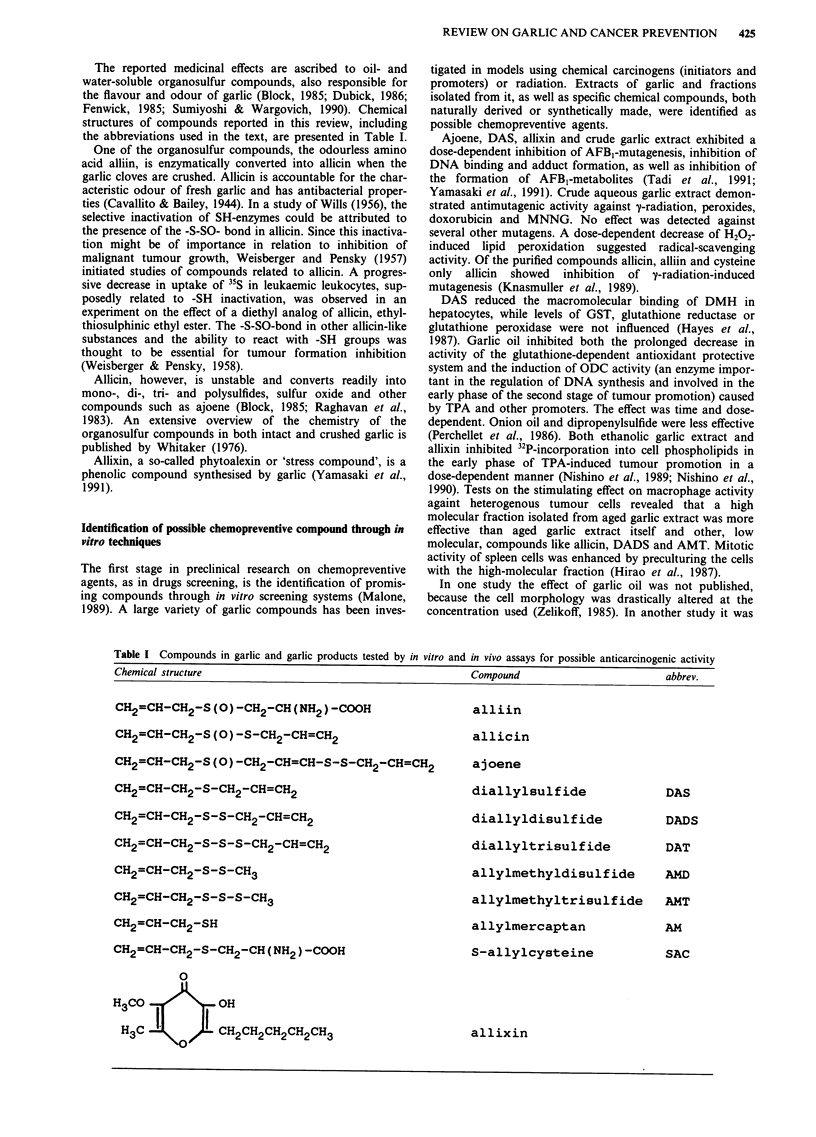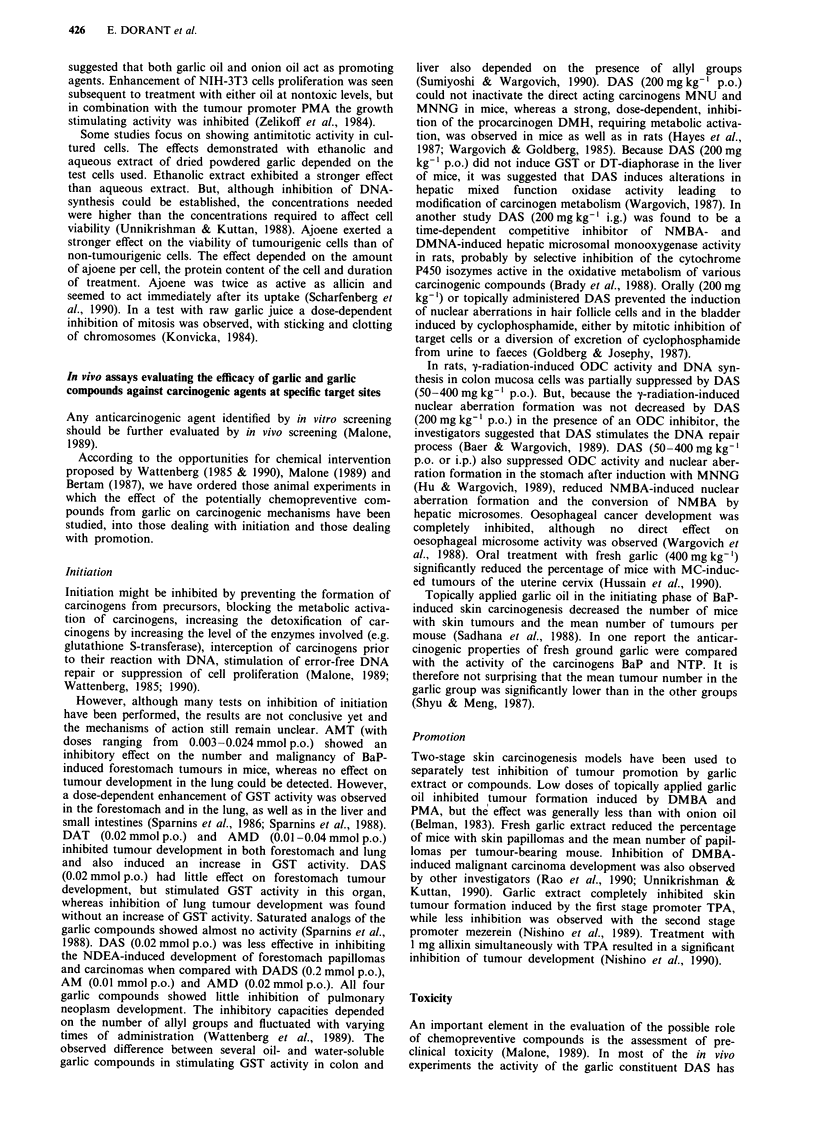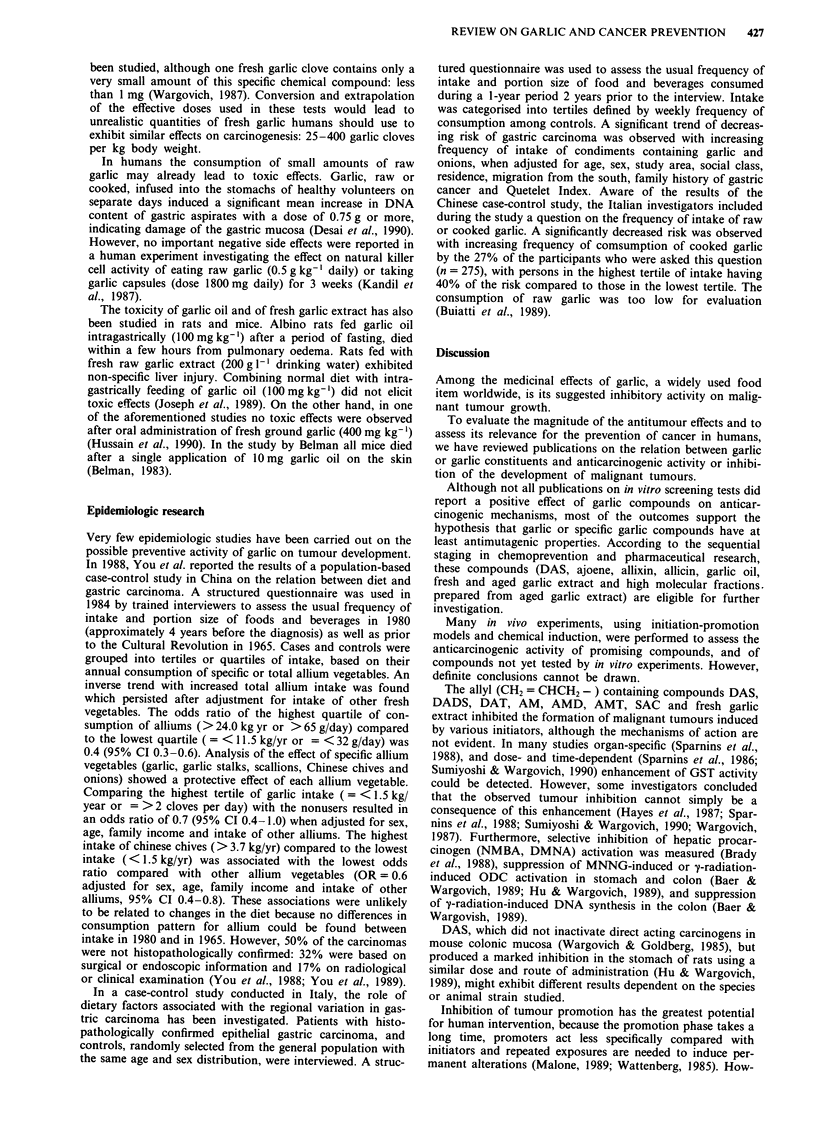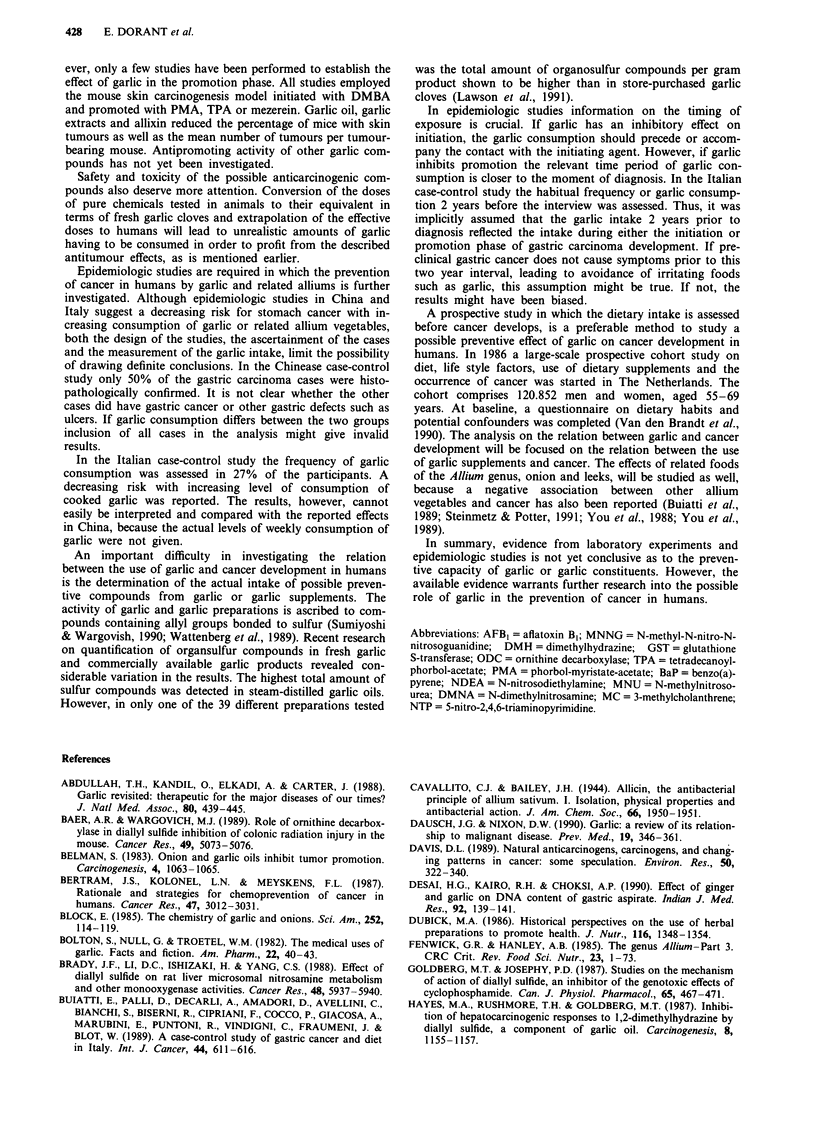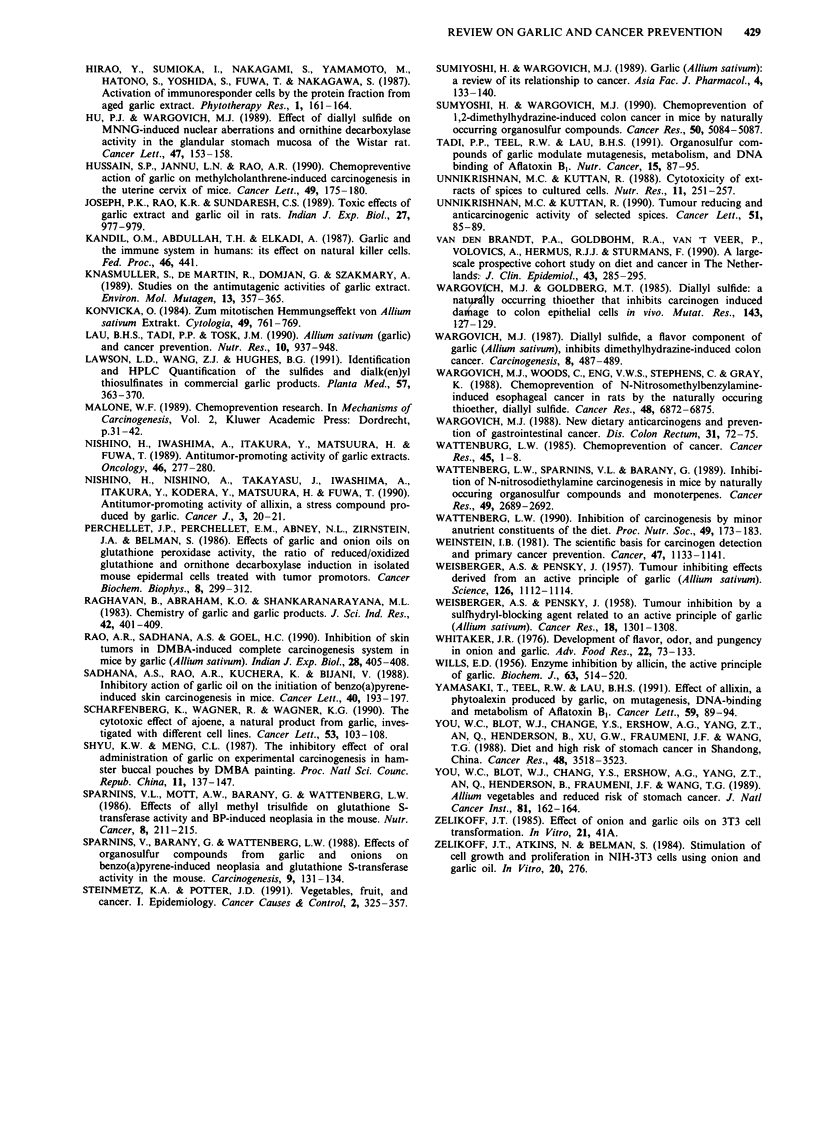# Garlic and its significance for the prevention of cancer in humans: a critical view.

**DOI:** 10.1038/bjc.1993.82

**Published:** 1993-03

**Authors:** E. Dorant, P. A. van den Brandt, R. A. Goldbohm, R. J. Hermus, F. Sturmans

**Affiliations:** Department of Epidemiology, University of Limburg, Maastricht.

## Abstract

Recently published results of epidemiologic case-control studies in China and Italy on gastric carcinoma in relation to diet suggest that consuming garlic may reduce the risk of gastric cancer. Chemical constituents of garlic have been tested for their inhibiting effect on carcinogenesis, using in vitro and in vivo models. In most experiments inhibition of tumour growth was established using fresh garlic extract, garlic compounds or synthetically prepared analogs. In this review the strengths and weaknesses of the experiments are discussed and the outcomes are evaluated to assess the possible significance of garlic or garlic compounds for the prevention of cancer in humans. It is concluded that evidence from laboratory experiments and epidemiologic studies is presently not conclusive as to the preventive activity of garlic. However, the available evidence warrants further research into the possible role of garlic in the prevention of cancer in humans.


*Br. J. Cancer* (1993), **67**, 424–429 © Macmillan Press Ltd., 1993

**REVIEW**

### Garlic and its significance for the prevention of cancer in humans: a critical view

E. Dorant[1], P.A. van den Brandt[1], R.A. Goldbohm[1,2], R.J.J. Hermus[2] & F. Sturmans[1]

[1]*Department of Epidemiology, University of Limburg, PO Box 616, 6200 MD Maastricht;* [2]*Department of Nutrition, TNO Toxicology and Nutrition Institute, Zeist, The Netherlands.*

**Summary** Recently published results of epidemiologic case-control studies in China and Italy on gastric carcinoma in relation to diet suggest that consuming garlic may reduce the risk of gastric cancer. Chemical constituents of garlic have been tested for their inhibiting effect on carcinogenesis, using *in vitro* and *in vivo* models. In most experiments inhibition of tumour growth was established using fresh garlic extract, garlic compounds or synthetically prepared analogs. In this review the strengths and weaknesses of the experiments are discussed and the outcomes are evaluated to assess the possible significance of garlic or garlic compounds for the prevention of cancer in humans. It is concluded that evidence from laboratory experiments and epidemiologic studies is presently not conclusive as to the preventive activity of garlic. However, the available evidence warrants further research into the possible role of garlic in the prevention of cancer in humans.

Elimination of carcinogenic substances from the environment is the preferable method to prevent malignant tumour development. However, contact with carcinogenic substances is not always avoidable (Wattenberg, 1985; Weinstein, 1981). Therefore, chemoprevention, in which the occurrence of cancer is prevented by administration of inhibitory compounds, has received increased attention (Malone, 1989; Wattenberg, 1985).

The relation between the use of allium vegetables, especially garlic (*Allium sativum*, a member of the genus *Allium* to which some 500 species belong), and its constituents, and the occurrence of cancer in humans is of particular interest in this respect. In a number of reviews on the effects of garlic on health, possible preventive effects on the development of cancer in humans have been mentioned. Fenwick and Hanley (1985) reviewed experimental studies designed to demonstrate any anticancer activity of garlic, together with the results of an epidemiological study in China in which death rates between two adjacent provinces were compared. In the latter study the highest gastric death rate had been found in the province with the lowest intake. The possible anticancer activity of garlic was illustrated in an earlier review of two studies reporting the inhibition of transplanted tumours in mice (Bolton *et al.*, 1982). In a publication on new dietary anticarcinogens and the prevention of gastrointestinal cancer, diallylsulfide, a component of garlic, was identified as a suppressing agent in dimethylhydrazine (DMH)-induced colon and nitrosomethylbenzylamine (NMBA)-induced oesophageal cancer development in rats (Wargovich, 1988). Suggested inhibitory mechanisms were evaluated in a review of selected recent publications on the effects of garlic on tumour formation in experimental animals (Sumiyoshi & Wargovich, 1989). In an earlier review free radical scavenging activity, immune system modulation and direct cytotoxic effect on cancer cells were discussed (Abdullah *et al.*, 1988). More recently the possible effects of garlic on detoxification systems *in vivo* and *in vitro* were reviewed (Dausch & Nixon, 1990). The changing patterns of cancer in the United States, United Kingdom and the Federal Republic of Germany led to speculations on the role of synthetic and natural carcinogens and anticarcinogens. Compounds of garlic were mentioned as inhibitors of tumour promotion (Davis, 1989). Based on the results from experimental and epidemiologic studies, Lau *et al.* (1990) concluded that garlic may be categorised as a dietary anticarcinogen.

Several investigators proposed a categorisation of substances with possible anticarcinogenic activity based on the steps in chemical induction of neoplasia (Bertram *et al.*, 1987; Malone, 1989; Wattenberg, 1985; 1990). In contrast with earlier reviews, we have ordered the *in vitro* and *in vivo* screening tests on the possible anticarcinogenic effects of garlic and garlic constituents according to this categorisation. Strengths and weaknesses of the experiments are discussed and whether their results provide any basis for the suggestion that garlic can prevent the development of cancer in humans.

Correspondence: E. Dorant, University of Limburg, Department of Epidemiology, PO Box 616, 6200 MD Maastricht, The Netherlands.
Received 16 April 1992; and in revised form 19 October 1992.

### Methods

Publications on garlic or garlic compounds and the relation with cancer, carcinogenicity or anticarcinogenicity were found by searching MEDLINE, a computer service available on CD-ROM (1983–1991) and by checking references to find earlier reports.

After a short overview of the chemical composition and active compounds of garlic, studies are reviewed on garlic preparations or specific chemical compounds possibly related to the proposed antitumour effect. According to the components of a sequential staging in preclinical research on chemopreventive agents (Malone, 1989), *in vitro* experiments on the identification of promising compounds are reviewed first. Next, *in vivo* screening tests involving animal models to evaluate the efficacy of compounds against carcinogenic agents at specific target sites, are reviewed.

Studies employing models in which malignant tumours are transplanted into experimental animals or models using spontaneous tumour formation are not reviewed, because these models are not considered relevant for research on cancer prevention (Malone, 1989).

Finally, the results of epidemiological studies in human populations are summarised and suggestions for further research are given.

### Chemical composition and active compounds of garlic

The main components of fresh garlic are water, carbohydrates, protein, fibre and fat. Garlic contains essential amino acids, vitamins and minerals. Garlic oil obtained by steam distillation, dehydrated garlic powder, pickled garlic, garlic juice and garlic extracts are available as condiments or nutritional supplements (Raghavan *et al.*, 1983). 'Aged garlic extract', a special garlic preparation, is obtained by extracting sliced raw garlic in a low concentration of ethanol at room temperature over a long period of time (Hirao *et al.*, 1987).

The reported medicinal effects are ascribed to oil- and water-soluble organosulfur compounds, also responsible for the flavour and odour of garlic (Block, 1985; Dubick, 1986; Fenwick, 1985; Sumiyoshi & Wargovich, 1990). Chemical structures of compounds reported in this review, including the abbreviations used in the text, are presented in Table I.

One of the organosulfur compounds, the odourless amino acid alliin, is enzymatically converted into allicin when the garlic cloves are crushed. Allicin is accountable for the characteristic odour of fresh garlic and has antibacterial properties (Cavallito & Bailey, 1944). In a study of Wills (1956), the selective inactivation of SH-enzymes could be attributed to the presence of the -S-SO- bond in allicin. Since this inactivation might be of importance in relation to inhibition of malignant tumour growth, Weisberger and Pensky (1957) initiated studies of compounds related to allicin. A progressive decrease in uptake of $^{35}S$ in leukaemic leukocytes, supposedly related to -SH inactivation, was observed in an experiment on the effect of a diethyl analog of allicin, ethylthiosulphinic ethyl ester. The -S-SO-bond in other allicin-like substances and the ability to react with -SH groups was thought to be essential for tumour formation inhibition (Weisberger & Pensky, 1958).

Allicin, however, is unstable and converts readily into mono-, di-, tri- and polysulfides, sulfur oxide and other compounds such as ajoene (Block, 1985; Raghavan *et al.*, 1983). An extensive overview of the chemistry of the organosulfur compounds in both intact and crushed garlic is published by Whitaker (1976).

Allixin, a so-called phytoalexin or 'stress compound', is a phenolic compound synthesised by garlic (Yamasaki *et al.*, 1991).

### Identification of possible chemopreventive compound through *in vitro* techniques

The first stage in preclinical research on chemopreventive agents, as in drugs screening, is the identification of promising compounds through *in vitro* screening systems (Malone, 1989). A large variety of garlic compounds has been investigated in models using chemical carcinogens (initiators and promoters) or radiation. Extracts of garlic and fractions isolated from it, as well as specific chemical compounds, both naturally derived or synthetically made, were identified as possible chemopreventive agents.

Ajoene, DAS, allixin and crude garlic extract exhibited a dose-dependent inhibition of $AFB_1$-mutagenesis, inhibition of DNA binding and adduct formation, as well as inhibition of the formation of $AFB_1$-metabolites (Tadi *et al.*, 1991; Yamasaki *et al.*, 1991). Crude aqueous garlic extract demonstrated antimutagenic activity against γ-radiation, peroxides, doxorubicin and MNNG. No effect was detected against several other mutagens. A dose-dependent decrease of $H_2O_2$-induced lipid peroxidation suggested radical-scavenging activity. Of the purified compounds allicin, alliin and cysteine only allicin showed inhibition of γ-radiation-induced mutagenesis (Knasmuller *et al.*, 1989).

DAS reduced the macromolecular binding of DMH in hepatocytes, while levels of GST, glutathione reductase or glutathione peroxidase were not influenced (Hayes *et al.*, 1987). Garlic oil inhibited both the prolonged decrease in activity of the glutathione-dependent antioxidant protective system and the induction of ODC activity (an enzyme important in the regulation of DNA synthesis and involved in the early phase of the second stage of tumour promotion) caused by TPA and other promoters. The effect was time and dose-dependent. Onion oil and dipropenylsulfide were less effective (Perchellet *et al.*, 1986). Both ethanolic garlic extract and allixin inhibited $^{32}P$-incorporation into cell phospholipids in the early phase of TPA-induced tumour promotion in a dose-dependent manner (Nishino *et al.*, 1989; Nishino *et al.*, 1990). Tests on the stimulating effect on macrophage activity againt heterogenous tumour cells revealed that a high molecular fraction isolated from aged garlic extract was more effective than aged garlic extract itself and other, low molecular, compounds like allicin, DADS and AMT. Mitotic activity of spleen cells was enhanced by preculturing the cells with the high-molecular fraction (Hirao *et al.*, 1987).

In one study the effect of garlic oil was not published, because the cell morphology was drastically altered at the concentration used (Zelikoff, 1985). In another study it was

**Table I** Compounds in garlic and garlic products tested by *in vitro* and *in vivo* assays for possible anticarcinogenic activity

| *Chemical structure* | *Compound* | *abbrev.* |
|---|---|---|
| $CH_2$=CH-$CH_2$-S(O)-$CH_2$-CH($NH_2$)-COOH | alliin | |
| $CH_2$=CH-$CH_2$-S(O)-S-$CH_2$-CH=$CH_2$ | allicin | |
| $CH_2$=CH-$CH_2$-S(O)-$CH_2$-CH=CH-S-S-$CH_2$-CH=$CH_2$ | ajoene | |
| $CH_2$=CH-$CH_2$-S-$CH_2$-CH=$CH_2$ | diallylsulfide | DAS |
| $CH_2$=CH-$CH_2$-S-S-$CH_2$-CH=$CH_2$ | diallyldisulfide | DADS |
| $CH_2$=CH-$CH_2$-S-S-S-$CH_2$-CH=$CH_2$ | diallyltrisulfide | DAT |
| $CH_2$=CH-$CH_2$-S-S-$CH_3$ | allylmethyldisulfide | AMD |
| $CH_2$=CH-$CH_2$-S-S-S-$CH_3$ | allylmethyltrisulfide | AMT |
| $CH_2$=CH-$CH_2$-SH | allylmercaptan | AM |
| $CH_2$=CH-$CH_2$-S-$CH_2$-CH($NH_2$)-COOH | S-allylcysteine | SAC |
|  | allixin | |

suggested that both garlic oil and onion oil act as promoting agents. Enhancement of NIH-3T3 cells proliferation was seen subsequent to treatment with either oil at nontoxic levels, but in combination with the tumour promoter PMA the growth stimulating activity was inhibited (Zelikoff *et al.*, 1984).

Some studies focus on showing antimitotic activity in cultured cells. The effects demonstrated with ethanolic and aqueous extract of dried powdered garlic depended on the test cells used. Ethanolic extract exhibited a stronger effect than aqueous extract. But, although inhibition of DNA-synthesis could be established, the concentrations needed were higher than the concentrations required to affect cell viability (Unnikrishman & Kuttan, 1988). Ajoene exerted a stronger effect on the viability of tumourigenic cells than of non-tumourigenic cells. The effect depended on the amount of ajoene per cell, the protein content of the cell and duration of treatment. Ajoene was twice as active as allicin and seemed to act immediately after its uptake (Scharfenberg *et al.*, 1990). In a test with raw garlic juice a dose-dependent inhibition of mitosis was observed, with sticking and clotting of chromosomes (Konvicka, 1984).

### *In vivo* assays evaluating the efficacy of garlic and garlic compounds against carcinogenic agents at specific target sites

Any anticarcinogenic agent identified by *in vitro* screening should be further evaluated by *in vivo* screening (Malone, 1989).

According to the opportunities for chemical intervention proposed by Wattenberg (1985 & 1990), Malone (1989) and Bertam (1987), we have ordered those animal experiments in which the effect of the potentially chemopreventive compounds from garlic on carcinogenic mechanisms have been studied, into those dealing with initiation and those dealing with promotion.

### *Initiation*

Initiation might be inhibited by preventing the formation of carcinogens from precursors, blocking the metabolic activation of carcinogens, increasing the detoxification of carcinogens by increasing the level of the enzymes involved (e.g. glutathione S-transferase), interception of carcinogens prior to their reaction with DNA, stimulation of error-free DNA repair or suppression of cell proliferation (Malone, 1989; Wattenberg, 1985; 1990).

However, although many tests on inhibition of initiation have been performed, the results are not conclusive yet and the mechanisms of action still remain unclear. AMT (with doses ranging from 0.003–0.024 mmol p.o.) showed an inhibitory effect on the number and malignancy of BaP-induced forestomach tumours in mice, whereas no effect on tumour development in the lung could be detected. However, a dose-dependent enhancement of GST activity was observed in the forestomach and in the lung, as well as in the liver and small intestines (Sparnins *et al.*, 1986; Sparnins *et al.*, 1988). DAT (0.02 mmol p.o.) and AMD (0.01–0.04 mmol p.o.) inhibited tumour development in both forestomach and lung and also induced an increase in GST activity. DAS (0.02 mmol p.o.) had little effect on forestomach tumour development, but stimulated GST activity in this organ, whereas inhibition of lung tumour development was found without an increase of GST activity. Saturated analogs of the garlic compounds showed almost no activity (Sparnins *et al.*, 1988). DAS (0.02 mmol p.o.) was less effective in inhibiting the NDEA-induced development of forestomach papillomas and carcinomas when compared with DADS (0.2 mmol p.o.), AM (0.01 mmol p.o.) and AMD (0.02 mmol p.o.). All four garlic compounds showed little inhibition of pulmonary neoplasm development. The inhibitory capacities depended on the number of allyl groups and fluctuated with varying times of administration (Wattenberg *et al.*, 1989). The observed difference between several oil- and water-soluble garlic compounds in stimulating GST activity in colon and liver also depended on the presence of allyl groups (Sumiyoshi & Wargovich, 1990). DAS (200 mg $kg^{-1}$ p.o.) could not inactivate the direct acting carcinogens MNU and MNNG in mice, whereas a strong, dose-dependent, inhibition of the procarcinogen DMH, requiring metabolic activation, was observed in mice as well as in rats (Hayes *et al.*, 1987; Wargovich & Goldberg, 1985). Because DAS (200 mg $kg^{-1}$ p.o.) did not induce GST or DT-diaphorase in the liver of mice, it was suggested that DAS induces alterations in hepatic mixed function oxidase activity leading to modification of carcinogen metabolism (Wargovich, 1987). In another study DAS (200 mg $kg^{-1}$ i.g.) was found to be a time-dependent competitive inhibitor of NMBA- and DMNA-induced hepatic microsomal monooxygenase activity in rats, probably by selective inhibition of the cytochrome P450 isozymes active in the oxidative metabolism of various carcinogenic compounds (Brady *et al.*, 1988). Orally (200 mg $kg^{-1}$) or topically administered DAS prevented the induction of nuclear aberrations in hair follicle cells and in the bladder induced by cyclophosphamide, either by mitotic inhibition of target cells or a diversion of excretion of cyclophosphamide from urine to faeces (Goldberg & Josephy, 1987).

In rats, γ-radiation-induced ODC activity and DNA synthesis in colon mucosa cells was partially suppressed by DAS (50–400 mg $kg^{-1}$ p.o.). But, because the γ-radiation-induced nuclear aberration formation was not decreased by DAS (200 mg $kg^{-1}$ p.o.) in the presence of an ODC inhibitor, the investigators suggested that DAS stimulates the DNA repair process (Baer & Wargovich, 1989). DAS (50–400 mg $kg^{-1}$ p.o. or i.p.) also suppressed ODC activity and nuclear aberration formation in the stomach after induction with MNNG (Hu & Wargovich, 1989), reduced NMBA-induced nuclear aberration formation and the conversion of NMBA by hepatic microsomes. Oesophageal cancer development was completely inhibited, although no direct effect on oesophageal microsome activity was observed (Wargovich *et al.*, 1988). Oral treatment with fresh garlic (400 mg $kg^{-1}$) significantly reduced the percentage of mice with MC-induced tumours of the uterine cervix (Hussain *et al.*, 1990).

Topically applied garlic oil in the initiating phase of BaP-induced skin carcinogenesis decreased the number of mice with skin tumours and the mean number of tumours per mouse (Sadhana *et al.*, 1988). In one report the anticarcinogenic properties of fresh ground garlic were compared with the activity of the carcinogens BaP and NTP. It is therefore not surprising that the mean tumour number in the garlic group was significantly lower than in the other groups (Shyu & Meng, 1987).

### *Promotion*

Two-stage skin carcinogenesis models have been used to separately test inhibition of tumour promotion by garlic extract or compounds. Low doses of topically applied garlic oil inhibited tumour formation induced by DMBA and PMA, but the effect was generally less than with onion oil (Belman, 1983). Fresh garlic extract reduced the percentage of mice with skin papillomas and the mean number of papillomas per tumour-bearing mouse. Inhibition of DMBA-induced malignant carcinoma development was also observed by other investigators (Rao *et al.*, 1990; Unnikrishman & Kuttan, 1990). Garlic extract completely inhibited skin tumour formation induced by the first stage promoter TPA, while less inhibition was observed with the second stage promoter mezerein (Nishino *et al.*, 1989). Treatment with 1 mg allixin simultaneously with TPA resulted in a significant inhibition of tumour development (Nishino *et al.*, 1990).

### Toxicity

An important element in the evaluation of the possible role of chemopreventive compounds is the assessment of preclinical toxicity (Malone, 1989). In most of the *in vivo* experiments the activity of the garlic constituent DAS has been studied, although one fresh garlic clove contains only a very small amount of this specific chemical compound: less than 1 mg (Wargovich, 1987). Conversion and extrapolation of the effective doses used in these tests would lead to unrealistic quantities of fresh garlic humans should use to exhibit similar effects on carcinogenesis: 25–400 garlic cloves per kg body weight.

In humans the consumption of small amounts of raw garlic may already lead to toxic effects. Garlic, raw or cooked, infused into the stomachs of healthy volunteers on separate days induced a significant mean increase in DNA content of gastric aspirates with a dose of 0.75 g or more, indicating damage of the gastric mucosa (Desai *et al.*, 1990). However, no important negative side effects were reported in a human experiment investigating the effect on natural killer cell activity of eating raw garlic (0.5 g $kg^{-1}$ daily) or taking garlic capsules (dose 1800 mg daily) for 3 weeks (Kandil *et al.*, 1987).

The toxicity of garlic oil and of fresh garlic extract has also been studied in rats and mice. Albino rats fed garlic oil intragastrically (100 mg $kg^{-1}$) after a period of fasting, died within a few hours from pulmonary oedema. Rats fed with fresh raw garlic extract (200 g $l^{-1}$ drinking water) exhibited non-specific liver injury. Combining normal diet with intragastrically feeding of garlic oil (100 mg $kg^{-1}$) did not elicit toxic effects (Joseph *et al.*, 1989). On the other hand, in one of the aforementioned studies no toxic effects were observed after oral administration of fresh ground garlic (400 mg $kg^{-1}$) (Hussain *et al.*, 1990). In the study by Belman all mice died after a single application of 10 mg garlic oil on the skin (Belman, 1983).

### Epidemiologic research

Very few epidemiologic studies have been carried out on the possible preventive activity of garlic on tumour development. In 1988, You *et al.* reported the results of a population-based case-control study in China on the relation between diet and gastric carcinoma. A structured questionnaire was used in 1984 by trained interviewers to assess the usual frequency of intake and portion size of foods and beverages in 1980 (approximately 4 years before the diagnosis) as well as prior to the Cultural Revolution in 1965. Cases and controls were grouped into tertiles or quartiles of intake, based on their annual consumption of specific or total allium vegetables. An inverse trend with increased total allium intake was found which persisted after adjustment for intake of other fresh vegetables. The odds ratio of the highest quartile of consumption of alliums (>24.0 kg yr or >65 g/day) compared to the lowest quartile ( = <11.5 kg/yr or = <32 g/day) was 0.4 (95% CI 0.3–0.6). Analysis of the effect of specific allium vegetables (garlic, garlic stalks, scallions, Chinese chives and onions) showed a protective effect of each allium vegetable. Comparing the highest tertile of garlic intake ( = <1.5 kg/ year or = >2 cloves per day) with the nonusers resulted in an odds ratio of 0.7 (95% CI 0.4–1.0) when adjusted for sex, age, family income and intake of other alliums. The highest intake of chinese chives (>3.7 kg/yr) compared to the lowest intake (<1.5 kg/yr) was associated with the lowest odds ratio compared with other allium vegetables (OR = 0.6 adjusted for sex, age, family income and intake of other alliums, 95% CI 0.4–0.8). These associations were unlikely to be related to changes in the diet because no differences in consumption pattern for allium could be found between intake in 1980 and in 1965. However, 50% of the carcinomas were not histopathologically confirmed: 32% were based on surgical or endoscopic information and 17% on radiological or clinical examination (You *et al.*, 1988; You *et al.*, 1989).

In a case-control study conducted in Italy, the role of dietary factors associated with the regional variation in gastric carcinoma has been investigated. Patients with histopathologically confirmed epithelial gastric carcinoma, and controls, randomly selected from the general population with the same age and sex distribution, were interviewed. A structured questionnaire was used to assess the usual frequency of intake and portion size of food and beverages consumed during a 1-year period 2 years prior to the interview. Intake was categorised into tertiles defined by weekly frequency of consumption among controls. A significant trend of decreasing risk of gastric carcinoma was observed with increasing frequency of intake of condiments containing garlic and onions, when adjusted for age, sex, study area, social class, residence, migration from the south, family history of gastric cancer and Quetelet Index. Aware of the results of the Chinese case-control study, the Italian investigators included during the study a question on the frequency of intake of raw or cooked garlic. A significantly decreased risk was observed with increasing frequency of comsumption of cooked garlic by the 27% of the participants who were asked this question ($n = 275$), with persons in the highest tertile of intake having 40% of the risk compared to those in the lowest tertile. The consumption of raw garlic was too low for evaluation (Buiatti *et al.*, 1989).

### Discussion

Among the medicinal effects of garlic, a widely used food item worldwide, is its suggested inhibitory activity on malignant tumour growth.

To evaluate the magnitude of the antitumour effects and to assess its relevance for the prevention of cancer in humans, we have reviewed publications on the relation between garlic or garlic constituents and anticarcinogenic activity or inhibition of the development of malignant tumours.

Although not all publications on *in vitro* screening tests did report a positive effect of garlic compounds on anticarcinogenic mechanisms, most of the outcomes support the hypothesis that garlic or specific garlic compounds have at least antimutagenic properties. According to the sequential staging in chemoprevention and pharmaceutical research, these compounds (DAS, ajoene, allixin, allicin, garlic oil, fresh and aged garlic extract and high molecular fractions. prepared from aged garlic extract) are eligible for further investigation.

Many *in vivo* experiments, using initiation-promotion models and chemical induction, were performed to assess the anticarcinogenic activity of promising compounds, and of compounds not yet tested by *in vitro* experiments. However, definite conclusions cannot be drawn.

The allyl ($CH_2 = CHCH_2-$) containing compounds DAS, DADS, DAT, AM, AMD, AMT, SAC and fresh garlic extract inhibited the formation of malignant tumours induced by various initiators, although the mechanisms of action are not evident. In many studies organ-specific (Sparnins *et al.*, 1988), and dose- and time-dependent (Sparnins *et al.*, 1986; Sumiyoshi & Wargovich, 1990) enhancement of GST activity could be detected. However, some investigators concluded that the observed tumour inhibition cannot simply be a consequence of this enhancement (Hayes *et al.*, 1987; Sparnins *et al.*, 1988; Sumiyoshi & Wargovich, 1990; Wargovich, 1987). Furthermore, selective inhibition of hepatic procarcinogen (NMBA, DMNA) activation was measured (Brady *et al.*, 1988), suppression of MNNG-induced or γ-radiation-induced ODC activation in stomach and colon (Baer & Wargovich, 1989; Hu & Wargovich, 1989), and suppression of γ-radiation-induced DNA synthesis in the colon (Baer & Wargovish, 1989).

DAS, which did not inactivate direct acting carcinogens in mouse colonic mucosa (Wargovich & Goldberg, 1985), but produced a marked inhibition in the stomach of rats using a similar dose and route of administration (Hu & Wargovich, 1989), might exhibit different results dependent on the species or animal strain studied.

Inhibition of tumour promotion has the greatest potential for human intervention, because the promotion phase takes a long time, promoters act less specifically compared with initiators and repeated exposures are needed to induce permanent alterations (Malone, 1989; Wattenberg, 1985). However, only a few studies have been performed to establish the effect of garlic in the promotion phase. All studies employed the mouse skin carcinogenesis model initiated with DMBA and promoted with PMA, TPA or mezerein. Garlic oil, garlic extracts and allixin reduced the percentage of mice with skin tumours as well as the mean number of tumours per tumour-bearing mouse. Antipromoting activity of other garlic compounds has not yet been investigated.

Safety and toxicity of the possible anticarcinogenic compounds also deserve more attention. Conversion of the doses of pure chemicals tested in animals to their equivalent in terms of fresh garlic cloves and extrapolation of the effective doses to humans will lead to unrealistic amounts of garlic having to be consumed in order to profit from the described antitumour effects, as is mentioned earlier.

Epidemiologic studies are required in which the prevention of cancer in humans by garlic and related alliums is further investigated. Although epidemiologic studies in China and Italy suggest a decreasing risk for stomach cancer with increasing consumption of garlic or related allium vegetables, both the design of the studies, the ascertainment of the cases and the measurement of the garlic intake, limit the possibility of drawing definite conclusions. In the Chinease case-control study only 50% of the gastric carcinoma cases were histopathologically confirmed. It is not clear whether the other cases did have gastric cancer or other gastric defects such as ulcers. If garlic consumption differs between the two groups inclusion of all cases in the analysis might give invalid results.

In the Italian case-control study the frequency of garlic consumption was assessed in 27% of the participants. A decreasing risk with increasing level of consumption of cooked garlic was reported. The results, however, cannot easily be interpreted and compared with the reported effects in China, because the actual levels of weekly consumption of garlic were not given.

An important difficulty in investigating the relation between the use of garlic and cancer development in humans is the determination of the actual intake of possible preventive compounds from garlic or garlic supplements. The activity of garlic and garlic preparations is ascribed to compounds containing allyl groups bonded to sulfur (Sumiyoshi & Wargovish, 1990; Wattenberg et al., 1989). Recent research on quantification of organsulfur compounds in fresh garlic and commercially available garlic products revealed considerable variation in the results. The highest total amount of sulfur compounds was detected in steam-distilled garlic oils. However, in only one of the 39 different preparations tested was the total amount of organosulfur compounds per gram product shown to be higher than in store-purchased garlic cloves (Lawson et al., 1991).

In epidemiologic studies information on the timing of exposure is crucial. If garlic has an inhibitory effect on initiation, the garlic consumption should precede or accompany the contact with the initiating agent. However, if garlic inhibits promotion the relevant time period of garlic consumption is closer to the moment of diagnosis. In the Italian case-control study the habitual frequency or garlic consumption 2 years before the interview was assessed. Thus, it was implicitly assumed that the garlic intake 2 years prior to diagnosis reflected the intake during either the initiation or promotion phase of gastric carcinoma development. If preclinical gastric cancer does not cause symptoms prior to this two year interval, leading to avoidance of irritating foods such as garlic, this assumption might be true. If not, the results might have been biased.

A prospective study in which the dietary intake is assessed before cancer develops, is a preferable method to study a possible preventive effect of garlic on cancer development in humans. In 1986 a large-scale prospective cohort study on diet, life style factors, use of dietary supplements and the occurrence of cancer was started in The Netherlands. The cohort comprises 120.852 men and women, aged 55–69 years. At baseline, a questionnaire on dietary habits and potential confounders was completed (Van den Brandt et al., 1990). The analysis on the relation between garlic and cancer development will be focused on the relation between the use of garlic supplements and cancer. The effects of related foods of the *Allium* genus, onion and leeks, will be studied as well, because a negative association between other allium vegetables and cancer has also been reported (Buiatti et al., 1989; Steinmetz & Potter, 1991; You et al., 1988; You et al., 1989).

In summary, evidence from laboratory experiments and epidemiologic studies is not yet conclusive as to the preventive capacity of garlic or garlic constituents. However, the available evidence warrants further research into the possible role of garlic in the prevention of cancer in humans.

Abbreviations: $AFB_1$ = aflatoxin $B_1$; MNNG = N-methyl-N-nitro-N-nitrosoguanidine; DMH = dimethylhydrazine; GST = glutathione S-transferase; ODC = ornithine decarboxylase; TPA = tetradecanoyl-phorbol-acetate; PMA = phorbol-myristate-acetate; BaP = benzo(a)-pyrene; NDEA = N-nitrosodiethylamine; MNU = N-methylnitroso-urea; DMNA = N-dimethylnitrosamine; MC = 3-methylcholanthrene; NTP = 5-nitro-2,4,6-triaminopyrimidine.

### References

ABDULLAH, T.H., KANDIL, O., ELKADI, A. & CARTER, J. (1988). Garlic revisited: therapeutic for the major diseases of our times? *J. Natl Med. Assoc.*, **80**, 439–445.

BAER, A.R. & WARGOVICH, M.J. (1989). Role of ornithine decarboxylase in diallyl sulfide inhibition of colonic radiation injury in the mouse. *Cancer Res.*, **49**, 5073–5076.

BELMAN, S. (1983). Onion and garlic oils inhibit tumor promotion. *Carcinogenesis*, **4**, 1063–1065.

BERTRAM, J.S., KOLONEL, L.N. & MEYSKENS, F.L. (1987). Rationale and strategies for chemoprevention of cancer in humans. *Cancer Res.*, **47**, 3012–3031.

BLOCK, E. (1985). The chemistry of garlic and onions. *Sci. Am.*, **252**, 114–119.

BOLTON, S., NULL, G. & TROETEL, W.M. (1982). The medical uses of garlic. Facts and fiction. *Am. Pharm.*, **22**, 40–43.

BRADY, J.F., LI, D.C., ISHIZAKI, H. & YANG, C.S. (1988). Effect of diallyl sulfide on rat liver microsomal nitrosamine metabolism and other monooxygenase activities. *Cancer Res.*, **48**, 5937–5940.

BUIATTI, E., PALLI, D., DECARLI, A., AMADORI, D., AVELLINI, C., BIANCHI, S., BISERNI, R., CIPRIANI, F., COCCO, P., GIACOSA, A., MARUBINI, E., PUNTONI, R., VINDIGNI, C., FRAUMENI, J. & BLOT, W. (1989). A case-control study of gastric cancer and diet in Italy. *Int. J. Cancer*, **44**, 611–616.

CAVALLITO, C.J. & BAILEY, J.H. (1944). Allicin, the antibacterial principle of allium sativum. I. Isolation, physical properties and antibacterial action. *J. Am. Chem. Soc.*, **66**, 1950–1951.

DAUSCH, J.G. & NIXON, D.W. (1990). Garlic: a review of its relationship to malignant disease. *Prev. Med.*, **19**, 346–361.

DAVIS, D.L. (1989). Natural anticarcinogens, carcinogens, and changing patterns in cancer: some speculation. *Environ. Res.*, **50**, 322–340.

DESAI, H.G., KAIRO, R.H. & CHOKSI, A.P. (1990). Effect of ginger and garlic on DNA content of gastric aspirate. *Indian J. Med. Res.*, **92**, 139–141.

DUBICK, M.A. (1986). Historical perspectives on the use of herbal preparations to promote health. *J. Nutr.*, **116**, 1348–1354.

FENWICK, G.R. & HANLEY, A.B. (1985). The genus *Allium*–Part 3. CRC Crit. *Rev. Food Sci. Nutr.*, **23**, 1–73.

GOLDBERG, M.T. & JOSEPHY, P.D. (1987). Studies on the mechanism of action of diallyl sulfide, an inhibitor of the genotoxic effects of cyclophosphamide. *Can. J. Physiol. Pharmacol.*, **65**, 467–471.

HAYES, M.A., RUSHMORE, T.H. & GOLDBERG, M.T. (1987). Inhibition of hepatocarcinogenic responses to 1,2-dimethylhydrazine by diallyl sulfide, a component of garlic oil. *Carcinogenesis*, **8**, 1155–1157.

HIRAO, Y., SUMIOKA, I., NAKAGAMI, S., YAMAMOTO, M., HATONO, S., YOSHIDA, S., FUWA, T. & NAKAGAWA, S. (1987). Activation of immunoresponder cells by the protein fraction from aged garlic extract. *Phytotherapy Res.*, **1**, 161–164.

HU, P.J. & WARGOVICH, M.J. (1989). Effect of diallyl sulfide on MNNG-induced nuclear aberrations and ornithine decarboxylase activity in the glandular stomach mucosa of the Wistar rat. *Cancer Lett.*, **47**, 153–158.

HUSSAIN, S.P., JANNU, L.N. & RAO, A.R. (1990). Chemopreventive action of garlic on methylcholanthrene-induced carcinogenesis in the uterine cervix of mice. *Cancer Lett.*, **49**, 175–180.

JOSEPH, P.K., RAO, K.R. & SUNDARESH, C.S. (1989). Toxic effects of garlic extract and garlic oil in rats. *Indian J. Exp. Biol.*, **27**, 977–979.

KANDIL, O.M., ABDULLAH, T.H. & ELKADI, A. (1987). Garlic and the immune system in humans: its effect on natural killer cells. *Fed. Proc.*, **46**, 441.

KNASMULLER, S., DE MARTIN, R., DOMJAN, G. & SZAKMARY, A. (1989). Studies on the antimutagenic activities of garlic extract. *Environ. Mol. Mutagen*, **13**, 357–365.

KONVICKA, O. (1984). Zum mitotischen Hemmungseffekt von *Allium sativum* Extrakt. *Cytologia*, **49**, 761–769.

LAU, B.H.S., TADI, P.P. & TOSK, J.M. (1990). *Allium sativum* (garlic) and cancer prevention. *Nutr. Res.*, **10**, 937–948.

LAWSON, L.D., WANG, Z.J. & HUGHES, B.G. (1991). Identification and HPLC Quantification of the sulfides and dialk(en)yl thiosulfinates in commercial garlic products. *Planta Med.*, **57**, 363–370.

MALONE, W.F. (1989). Chemoprevention research. In *Mechanisms of Carcinogenesis*, Vol. 2, Kluwer Academic Press: Dordrecht, p.31–42.

NISHINO, H., IWASHIMA, A., ITAKURA, Y., MATSUURA, H. & FUWA, T. (1989). Antitumor-promoting activity of garlic extracts. *Oncology*, **46**, 277–280.

NISHINO, H., NISHINO, A., TAKAYASU, J., IWASHIMA, A., ITAKURA, Y., KODERA, Y., MATSUURA, H. & FUWA, T. (1990). Antitumor-promoting activity of allixin, a stress compound produced by garlic. *Cancer J.*, **3**, 20–21.

PERCHELLET, J.P., PERCHELLET, E.M., ABNEY, N.L., ZIRNSTEIN, J.A. & BELMAN, S. (1986). Effects of garlic and onion oils on glutathione peroxidase activity, the ratio of reduced/oxidized glutathione and ornithone decarboxylase induction in isolated mouse epidermal cells treated with tumor promotors. *Cancer Biochem. Biophys.*, **8**, 299–312.

RAGHAVAN, B., ABRAHAM, K.O. & SHANKARANARAYANA, M.L. (1983). Chemistry of garlic and garlic products. *J. Sci. Ind. Res.*, **42**, 401–409.

RAO, A.R., SADHANA, A.S. & GOEL, H.C. (1990). Inhibition of skin tumors in DMBA-induced complete carcinogenesis system in mice by garlic (*Allium sativum*). *Indian J. Exp. Biol.*, **28**, 405–408.

SADHANA, A.S., RAO, A.R., KUCHERA, K. & BIJANI, V. (1988). Inhibitory action of garlic oil on the initiation of benzo(a)pyrene-induced skin carcinogenesis in mice. *Cancer Lett.*, **40**, 193–197.

SCHARFENBERG, K., WAGNER, R. & WAGNER, K.G. (1990). The cytotoxic effect of ajoene, a natural product from garlic, investigated with different cell lines. *Cancer Lett.*, **53**, 103–108.

SHYU, K.W. & MENG, C.L. (1987). The inhibitory effect of oral administration of garlic on experimental carcinogenesis in hamster buccal pouches by DMBA painting. *Proc. Natl Sci. Counc. Repub. China*, **11**, 137–147.

SPARNINS, V.L., MOTT, A.W., BARANY, G. & WATTENBERG, L.W. (1986). Effects of allyl methyl trisulfide on glutathione S-transferase activity and BP-induced neoplasia in the mouse. *Nutr. Cancer*, **8**, 211–215.

SPARNINS, V., BARANY, G. & WATTENBERG, L.W. (1988). Effects of organosulfur compounds from garlic and onions on benzo(a)pyrene-induced neoplasia and glutathione S-transferase activity in the mouse. *Carcinogenesis*, **9**, 131–134.

STEINMETZ, K.A. & POTTER, J.D. (1991). Vegetables, fruit, and cancer. I. Epidemiology. *Cancer Causes & Control*, **2**, 325–357.

SUMIYOSHI, H. & WARGOVICH, M.J. (1989). Garlic (*Allium sativum*): a review of its relationship to cancer. *Asia Fac. J. Pharmacol.*, **4**, 133–140.

SUMYOSHI, H. & WARGOVICH, M.J. (1990). Chemoprevention of 1,2-dimethylhydrazine-induced colon cancer in mice by naturally occurring organosulfur compounds. *Cancer Res.*, **50**, 5084–5087.

TADI, P.P., TEEL, R.W. & LAU, B.H.S. (1991). Organosulfur compounds of garlic modulate mutagenesis, metabolism, and DNA binding of Aflatoxin $B_1$. *Nutr. Cancer*, **15**, 87–95.

UNNIKRISHNAN, M.C. & KUTTAN, R. (1988). Cytotoxicity of extracts of spices to cultured cells. *Nutr. Res.*, **11**, 251–257.

UNNIKRISHNAN, M.C. & KUTTAN, R. (1990). Tumour reducing and anticarcinogenic activity of selected spices. *Cancer Lett.*, **51**, 85–89.

VAN DEN BRANDT, P.A., GOLDBOHM, R.A., VAN 'T VEER, P., VOLOVICS, A., HERMUS, R.J.J. & STURMANS, F. (1990). A large-scale prospective cohort study on diet and cancer in The Netherlands. *J. Clin. Epidemiol.*, **43**, 285–295.

WARGOVICH, M.J. & GOLDBERG, M.T. (1985). Diallyl sulfide: a naturally occurring thioether that inhibits carcinogen induced damage to colon epithelial cells *in vivo*. *Mutat. Res.*, **143**, 127–129.

WARGOVICH, M.J. (1987). Diallyl sulfide, a flavor component of garlic (*Allium sativum*), inhibits dimethylhydrazine-induced colon cancer. *Carcinogenesis*, **8**, 487–489.

WARGOVICH, M.J., WOODS, C., ENG, V.W.S., STEPHENS, C. & GRAY, K. (1988). Chemoprevention of N-Nitrosomethylbenzylamine-induced esophageal cancer in rats by the naturally occuring thioether, diallyl sulfide. *Cancer Res.*, **48**, 6872–6875.

WARGOVICH, M.J. (1988). New dietary anticarcinogens and prevention of gastrointestinal cancer. *Dis. Colon Rectum*, **31**, 72–75.

WATTENBURG, L.W. (1985). Chemoprevention of cancer. *Cancer Res.*, **45**, 1–8.

WATTENBERG, L.W., SPARNINS, V.L. & BARANY, G. (1989). Inhibition of N-nitrosodiethylamine carcinogenesis in mice by naturally occuring organosulfur compounds and monoterpenes. *Cancer Res.*, **49**, 2689–2692.

WATTENBERG, L.W. (1990). Inhibition of carcinogenesis by minor anutrient constituents of the diet. *Proc. Nutr. Soc.*, **49**, 173–183.

WEINSTEIN, I.B. (1981). The scientific basis for carcinogen detection and primary cancer prevention. *Cancer*, **47**, 1133–1141.

WEISBERGER, A.S. & PENSKY, J. (1957). Tumour inhibiting effects derived from an active principle of garlic (*Allium sativum*). *Science*, **126**, 1112–1114.

WEISBERGER, A.S. & PENSKY, J. (1958). Tumour inhibition by a sulfhydryl-blocking agent related to an active principle of garlic (*Allium sativum*). *Cancer Res.*, **18**, 1301–1308.

WHITAKER, J.R. (1976). Development of flavor, odor, and pungency in onion and garlic. *Adv. Food Res.*, **22**, 73–133.

WILLS, E.D. (1956). Enzyme inhibition by allicin, the active principle of garlic. *Biochem. J.*, **63**, 514–520.

YAMASAKI, T., TEEL, R.W. & LAU, B.H.S. (1991). Effect of allixin, a phytoalexin produced by garlic, on mutagenesis, DNA-binding and metabolism of Aflatoxin $B_1$. *Cancer Lett.*, **59**, 89–94.

YOU, W.C., BLOT, W.J., CHANGE, Y.S., ERSHOW, A.G., YANG, Z.T., AN, Q., HENDERSON, B., XU, G.W., FRAUMENI, J.F. & WANG, T.G. (1988). Diet and high risk of stomach cancer in Shandong, China. *Cancer Res.*, **48**, 3518–3523.

YOU, W.C., BLOT, W.J., CHANG, Y.S., ERSHOW, A.G., YANG, Z.T., AN, Q., HENDERSON, B., FRAUMENI, J.F. & WANG, T.G. (1989). *Allium* vegetables and reduced risk of stomach cancer. *J. Natl Cancer Inst.*, **81**, 162–164.

ZELIKOFF, J.T. (1985). Effect of onion and garlic oils on 3T3 cell transformation. *In Vitro*, **21**, 41A.

ZELIKOFF, J.T., ATKINS, N. & BELMAN, S. (1984). Stimulation of cell growth and proliferation in NIH-3T3 cells using onion and garlic oil. *In Vitro*, **20**, 276.